# Mutation bias and the predictability of evolution

**DOI:** 10.1098/rstb.2022.0055

**Published:** 2023-05-22

**Authors:** Alejandro V. Cano, Bryan L. Gitschlag, Hana Rozhoňová, Arlin Stoltzfus, David M. McCandlish, Joshua L. Payne

**Affiliations:** ^1^ Institute of Integrative Biology, ETH Zurich, 8092 Zurich, Switzerland; ^2^ Swiss Institute of Bioinformatics, 1015 Lausanne, Switzerland; ^3^ Simons Center for Quantitative Biology, Cold Spring Harbor Laboratory, Cold Spring Harbor, NY 11724, USA; ^4^ Office of Data and Informatics, Material Measurement Laboratory, National Institute of Standards and Technology, Rockville, MD 20899, USA; ^5^ Institute for Bioscience and Biotechnology Research, Rockville, MD 20850, USA

**Keywords:** adaptation, mutation, prediction, theory, population genetics

## Abstract

Predicting evolutionary outcomes is an important research goal in a diversity of contexts. The focus of evolutionary forecasting is usually on adaptive processes, and efforts to improve prediction typically focus on selection. However, adaptive processes often rely on new mutations, which can be strongly influenced by predictable biases in mutation. Here, we provide an overview of existing theory and evidence for such mutation-biased adaptation and consider the implications of these results for the problem of prediction, in regard to topics such as the evolution of infectious diseases, resistance to biochemical agents, as well as cancer and other kinds of somatic evolution. We argue that empirical knowledge of mutational biases is likely to improve in the near future, and that this knowledge is readily applicable to the challenges of short-term prediction.

This article is part of the theme issue ‘Interdisciplinary approaches to predicting evolutionary biology’.

## 1. Introduction

Predicting the dynamics and outcome of evolution is an important goal of the biological sciences, offering the potential to design better drugs, combat pathogens and conserve endangered species [1–11]. Targets of prediction include genetic changes underlying adaptation, such as those causing antibiotic resistance or enhancing thermostability, as well as their corresponding phenotypes, such as minimum inhibitory concentration or melting temperature [12,13]. Higher-level targets include the diversity, abundance and ecosystem functions of microbial communities [14], as well as the rate of adaptation itself [13].

Owing to the stochastic nature of the evolutionary process, forecasting offers the greatest potential over short and intermediate timescales. Our ability to make accurate forecasts depends crucially on high-quality experimental data, such as those describing the phenotypic or fitness effects of mutations. For example, over short timescales, where one may wish to predict the next beneficial mutation to arise and go to fixation, empirical knowledge of the distribution of fitness effects is key, because this provides information about the fixation probabilities of new mutations [15]. At intermediate timescales, where one may wish to predict which of several possible mutational trajectories to adaptation is the most likely, empirical knowledge of the fitness effects of combinations of mutations is key, because this can be used to delineate between mutational trajectories that ascend adaptive peaks from those that fall into maladaptive valleys [2]. As such, the project of predicting evolution has benefited greatly from recent advances in high-throughput sequencing technologies and phenotypic assays, which ameliorate so-called ‘data limits’ on accurate forecasting [7]. These technologies have been used to characterize the phenotypic and fitness effects of mutations in a diversity of biological systems, including regulatory elements [16,17], macromolecules [18–24], gene regulatory circuits [25,26] and metabolic pathways [27].

However, empirical knowledge of the phenotypic and fitness effects of mutations only takes us so far. Whereas these data provide useful information about the likelihood of mutations going to fixation, they tell us nothing about the rate with which new mutations are introduced into a population. This is an important limitation, because evolution often proceeds via the introduction of new mutations, and some types of mutations are more likely to arise than others [28,29]. For example, studies of the rates and spectra of spontaneous mutations, such as those based on mutation accumulation experiments, have revealed a bias towards transitions (purine-to-purine or pyrimidine-to-pyrimidine changes), relative to transversions (purine-to-pyrimidine changes, or vice versa) in a wide range of species [30]. The exact degree of transition bias emerging under any particular set of conditions is the net outcome of biases in all stages in the genesis of nucleotide mutations, including biases in susceptibility to damage (e.g. oxidative damage), in the efficiency of damage recognition and repair, in rates of polymerase errors and proofreading, and in the efficiency of recognition and repair of mispaired bases (see [31,32]). Because transition bias and other kinds of mutation bias make some mutational steps to adaptation more likely than others, empirical knowledge of mutation bias offers the potential to improve evolutionary forecasting, both at short and intermediate timescales.

Here, we address how effects of mutational biases—predictable differences in rates between different categories of mutational conversions—make evolution more predictable, focusing mostly on the case of short-term adaptation from new mutations, and setting aside some related topics such as the role of specialized mutation-generating systems [33, ch. 5] and hypermutators [34]. First, we review theoretical work suggesting that such biases can exert a strong influence on the outcome of evolutionary processes, including adaptive processes, that depend on new mutations. Next, we review the empirical case for an influence of mutation bias on adaptation in the laboratory and in nature. Finally, we discuss specific applications where empirical knowledge of mutation bias is anticipated to improve evolutionary forecasting, in regard to topics such as infectious diseases, cancer and other kinds of somatic evolution, as well as resistance to biochemical agents. We note some recurring themes: (i) the most commonly observed outcome is often the most mutationally favourable of the adaptive options, not the most fit, (ii) ordinary nucleotide mutation biases often have strong and predictable effects on the genetic changes underlying adaptation, (iii) perturbing the mutation spectrum alters the distribution of such changes, and (iv) the influence of mutation biases can be altered by the beneficial mutation supply and other population-genetic and environmental conditions. In general, we argue that knowledge of mutation can improve predictability in practical contexts. We conclude with comments on open questions and future prospects.

## 2. Theory

Under what conditions will empirical knowledge of mutation bias improve evolutionary forecasting? To address this question, we first turn to theory. The classic ‘Modern Synthesis’ view assumes evolution from standing variation in an abundant gene pool, so that the process of evolution is formally a process of recombining and shifting frequencies of available alleles without new mutations [33,35,36]. In this context, adaptation happens by selectively favourable shifts in frequencies of multi-locus combinations of small-effect alleles generated by recombination [37–40]. The role of mutation is strictly limited: recurrent mutation acts only as a weak pressure, ineffectual except when mutation rates are high and unopposed by selection [41–43]. Therefore, in this theory, the predictability of evolution emerges from a consideration of selection: in the short-term, an evolving population ascends a fitness gradient in a multi-locus allele-frequency space; in the long term, it approaches a local or global maximum of fitness.

A different view of the roles of mutation and selection emerged during the molecular revolution. Comparisons of protein sequences suggested that evolutionary divergence occurs by the accumulation of individual substitutions of amino acid residues, where each substitution reflects a mutation that was promoted—or at least, tolerated—by selection, which was conceptualized as a filter acting on individual mutations [44–46]. This way of thinking placed the process of mutation in the more important role of offering individual variants directly for selective filtering (rather than merely filling up the gene pool to facilitate subsequent recombination). This conception of evolution as a two-step process was formalized in ‘origin-fixation’ models, which depict the limiting behaviour of evolution when the number of new mutations introduced per generation becomes arbitrarily small [47]. In an origin-fixation model, the rate of evolution is determined by the product of a rate of ‘introduction’ or origin *Nμ* and a probability of fixation *π*, i.e. *R* = *Nμπ*.

Importantly, this new way of thinking about evolution suggests an increased influence for mutation biases, because the likelihood of each possible step will depend on the likelihood of the underlying mutation. For evolution in the origin-fixation regime, mutational biases (i.e. biases in origination) and biases in fixation each have proportional effects on the course of evolution [29], i.e. we can express a ratio of origin-fixation rates in terms of these two different types of biases:

2.1 $$\frac{R_{ij}}{R_{ik}}=\frac{\mu_{ij}N\pi_{ij}}{\mu_{ik}N\pi_{ik}}=\frac{\mu_{ij}}{\mu_{ik}}\;*\;\frac{\pi_{ij}}{\pi_{ik}},$$

where *R*_*ij*_ is the rate of change from allele *i* to allele *j*, *μ*_*ij*_ is the mutation rate from allele *i* to allele *j*, *π*_*ij*_ is the chance of fixation of a new allele of type *j* in a population otherwise of type *i*, and *N* is the population size (see also [48]). That is, the evolutionary bias between two alternative types of changes, *i* → *j* versus *i* → *k*, can be expressed as the product of a bias in origination (e.g. transition-transversion bias or GC-AT bias) and a bias in fixation [29,48]. This means that biases in the introduction process can influence adaptation even when mutation rates are low and selection is strong, in contrast to the classical view in which internal biases are assumed to require evolution by mutation pressure [41–43], which requires high rates of mutation.

The equation above reflects origin-fixation conditions, and is useful for thinking about short-term evolution, or about long-term evolution in an infinite space. What about less ideal conditions, e.g. extended adaptive walks on finite landscapes? To grasp the potential effects of mutation bias on adaptive walks, it is helpful to consider the different perspectives of points, paths, local peaks and landscapes. From a typical point on a complex landscape, multiple upward (fitness-increasing) steps are possible, and some are mutationally favourable (whereas others are not), so that the orientation of an evolving system may be biased. Any path of upward steps eventually ends at some local peak, and some paths are enriched in mutationally favourable steps (whereas others are not), so that a system evolving under a bias may favour some paths over others. From the perspective of peaks, each fitness peak is accessible by some set of upward paths, and this set of paths may differ in size, and may be more or less enriched for mutationally favourable paths, so that certain peaks may be more likely outcomes of evolution, averaging over many possible starting points. Finally, for a given landscape with many peaks, we can define all the upward paths, i.e. all the possible adaptive walks, and thus some landscapes will have more mutationally favoured walks, making them more navigable.

Evolutionary simulations on complex adaptive landscapes confirm these broad expectations and provide some guidance on the size of effects [49–53]. For instance, [52] modelled adaptive walks using an NK model of fitness applied to a protein encoded by a gene subject to variable GC : AT bias, finding that a several-fold bias in mutation can have a substantial impact on the amino acid composition of evolved proteins. Cano & Payne [49] explored the effect of transition-transversion bias on the navigability of empirical landscapes for transcription-factor binding sites, finding that the landscapes are most navigable when the mutation bias matches the bias inherent in the landscape. Schaper & Louis [51] find that RNA folds with the most sequences are more findable in adaptation.

How far do effects of mutation biases extend outside of the strict origin-fixation regime that emerges as the mutation supply *μN* becomes arbitrarily small? In the hypothetical case of an infinite-sites model, mutation biases are influential regardless of mutation supply (appendix A). For finite cases, the results of Yampolsky & Stoltzfus [29] suggest that biases in the introduction process decay with mutation supply but remain influential well outside the origin-fixation regime. Subsequent work has clarified this relationship [56–58]. In particular, Cano *et al.* [56] used simulations to study the effect of mutation supply in a codon-based model of protein adaptation. They quantified the effect of mutation bias with a single statistic, *β*, which ranges from 0, indicating no influence, to 1, indicating that the spectrum of amino acid-changing mutations has a proportional influence on the spectrum of changes fixed in adaptation. They found that *β* ≈ 1 when the mutation supply is low (*Nμ* ≈ 10^−4^), and ultimately goes to 0 for high mutation supply, with most of the shift from 1 to 0 happening as mutation supply goes from 10^−2^ to 10^0^.

Finally, what are the implications for predictability? As explained in appendix B, considering a single adaptive step, predictability (in the sense of repeatability) decreases with the number of possibilities, and increases with the variance in their probabilities [63,66]. This predictability can be partitioned further (under limiting conditions explained in appendix B) into contributions of mutation and fixation. The separate terms have the same property that, the greater the variability in the probability of fixation *π*, or the greater the variability in *μ*, the greater the contribution to repeatability. An important implication of this theoretical result is that, in designing approaches to prediction, it is important to capture as much variance as possible in elementary chances, and to treat mutation and selection comparably to avoid a skewed picture of their contributions. For instance, if 40 different beneficial mutations are possible, and we use individual fitness measurements for each *s*, but characterize each *μ* with an average rate from a model of six types of rates, this artificially reduces the expected contribution of mutation to repeatability, given that such simplified models capture only a minority of the variance in individual mutation rates [67].

What about predictability in long-term adaptive walks? In the special case of adaptation on a fixed and finite landscape without epistasis, the evolving system will converge on a single global peak, and mutation bias will influence the trajectory and the length of the walk, but not the final destination. In any other case, mutation bias may influence the direction, length and ultimate destination of a walk, as outlined above. Predictability has a somewhat counterintuitive relationship to mutation bias when a system with a particular bias is on a landscape enriched for upward paths favoured by that bias. In this case, as shown by Cano & Payne [49], there is a larger set of upward paths enriched for mutationally favourable changes, and so the particular path taken in any instance of adaptation is less predictable.

Predicting evolutionary trajectories is further complicated by the potential for changes in the mutation spectrum itself, which can occur even on short timescales, owing to transient changes in environmental conditions [68,69]. Durable genetic changes in the mutation spectrum that may be important in evolution on various timescales include (i) the emergence of hypermutators with greatly enhanced mutation rates and distinct mutation spectra [34,70], (ii) changes that modify mutation spectra without dramatic changes in total mutation rate [71,72], (iii) long-term changes in DNA repaire repertoire including the loss and gain of entire pathways [73], (iv) shifts in (and long-term equilibration of) the genomic frequency of sequence contexts under the long-continued influence of context-dependent mutation [74], (v) genome-wide patterns of adaptive amelioration reducing the frequency or severity of deleterious mutations [75,76], and (vi) bias reversals that temporarily enhance the rate of adaptation by enhancing mutational access to previously under-sampled classes of beneficial mutations [50,53].

In summary, theory suggests that mutation bias can influence adaptation under a broad range of population genetic conditions, with the strongest signal of mutational influence appearing when the mutation supply is low. Mutation biases can influence both the outcomes of short-term adaptation, and the trajectory, length, and outcome of adaptive walks, dependent on conditions. The extent to which empirical knowledge of mutation bias will improve evolutionary forecasting depends on the extent to which natural systems evolve under conditions favourable to these effects. Because this is an empirical issue, we next turn to experimental evidence, from the laboratory and nature.

## 3. Evidence

As outlined above, theory suggests that, where conditions allow, systematic biases in mutation can shape the course of adaptation via biases in the introduction process. What is the evidence that this kind of causation is real? What do we know about effect-sizes under various conditions? How well do these effects fit theoretical expectations? How broadly are such effects expected?

### (a) Causal agency

To begin, one may ask what studies establish causal agency, i.e. proving beyond any reasonable doubt that X causes Y? The gold standard is to manipulate X and show the expected effects on Y under controlled conditions. This standard is satisfied by the work of Couce *et al.* [77] and Horton *et al.* [78], laboratory studies with microbial systems, involving adaptation from new mutations under controlled conditions that include direct manipulation of the mutation spectrum.

**Figure 1.**
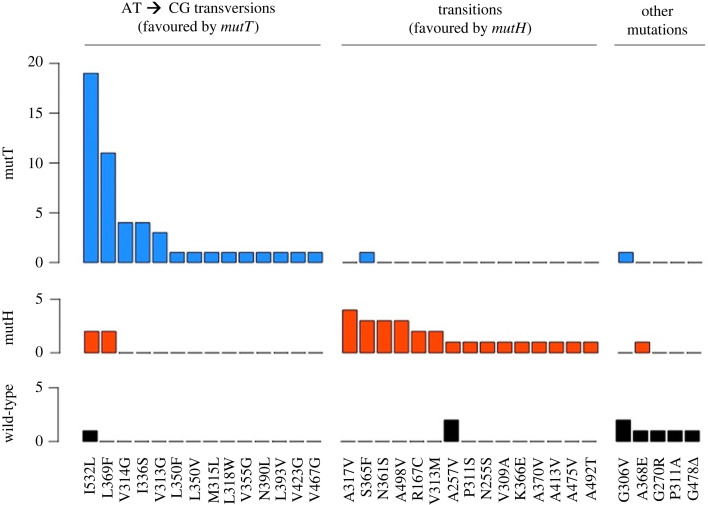
The mutation spectrum shapes adaptation. Cefotaxime-resistant variants from a *mutT* parent (top row, blue) tend to have the kinds of mutations favoured by *mutT* (left block of columns, *A* : *T* > *C* : *G* transversions), and likewise for resistant variants from a *mutH* parent (middle row, red) which tend to have the transitions (middle block of columns) favoured by *mutH*. Data for *ftsI* variants provided by Alejandro Couce.

Couce *et al.* [77] subjected 192 replicate lines of *Escherichia coli* to increasing concentrations of the *β*-lactam antibiotic cefotaxime, using three different parental strains: wild-type, *mutH* and *mutT*. The latter two are mutators with higher overall rates of mutation and distinctive biases toward transitions (*mutH*) or *A* : *T* → *C* : *G* transversions (*mutT*). Figure 1 shows the resistance-conferring mutations that arose in *ftsI*, the gene in which most of these mutations are found. The resistance-conferring mutations from *mutT* isolates (blue) tend to be *A* : *T* → *C* : *G* transversions (left block of bars), which are the type favoured by *mutT*, whereas the resistance-conferring mutations that evolved in the *mutH* strain (red) tend to be the transitions (centre block of bars) favoured by *mutH*. That is, changing the mutation spectrum changes the spectrum of adaptive changes in a corresponding manner.

The second study, by Horton *et al.* [78], was motivated by the observation that two different strains of *Pseudomonas fluorescens* adapt to the loss of motility in strikingly different ways. In one strain, over 95 per cent of the time, adaptation involved an A289C change in the *ntrB* locus, whereas in the other strain, adaptation involved mutations in diverse genes. They identified a hotspot mutation associated with synonymous sequence differences in the two strains. To test that the mutational hotspot caused the difference in adaptation, they used genetic engineering to create the hotspot in one strain, and remove it in the other—all without changing the protein sequence (because the engineered changes were synonymous). The results confirmed the mutational hypothesis. When the hotspot was removed, adaptation no longer relied on the mutation in the *ntrB* locus; and when the hotspot was engineered, adaptation no longer involved mutations in diverse genes, but rather relied on the A289C mutation.

### (b) Range of effect-sizes

Having established causal agency with studies that involve unusual conditions—some mutators and a hotspot—let us now ask about effect-sizes when ordinary nucleotide mutation biases are involved, and particularly, let us consider whether quantitative relationships between *s*, *μ* and the frequency with which a variant evolves *f* are roughly what we expect from theory. Several studies are useful in this regard. We will focus here on Maclean *et al.* [62], Rokyta *et al.* [59] and Cano *et al.* [56].

**Figure 2.**
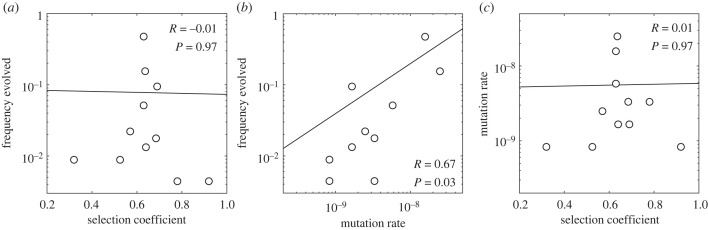
Inter-relations of mutation rate, selection coefficient and frequency evolved in a laboratory adaptation experiment (*R*, Pearson’s correlation coefficient; *P*, the *p*-value). From 284 replicate cultures adapted to Rifampicin, Maclean *et al.* [62] identified 35 different *rpoB* mutations, many emerging repeatedly, reporting their frequency of evolving (in replicate cultures), and reporting mutation rates for 11 of the variants (frequency evolved is the combined frequency in backgrounds where the mutation is possible, using data in table 1 from Maclean *et al.* [62]; selection coefficients are averaged when multiple values are reported for different backgrounds). In the three panels, linear regressions were performed on the unlogged values, and Pearson’s correlation coefficients and their respective *p*-values are shown.

Maclean *et al.* [62] tracked the emergence of resistance to Rifampicin in replicate cultures of *Pseudomonas aeruginosa*. Resistant strains typically have mutations in *rpoB*, encoding the main RNA polymerase subunit. Maclean *et al.* [62] measured selection coefficients for 35 resistant variants, and mutation rates for 11 of these. The mutation rates—all for single-nucleotide substitutions—ranged 30-fold. However, the selection coefficients are very large and show a much smaller range, from 0.3 to 0.9, so that the range expected for the probability of fixation is even smaller, just 0.45 to 0.83 (using the formula of [60]). Thus, under origin-fixation conditions, we expect a 30-fold effect of mutation but only about a twofold effect of selection (given that clonal interference is not expected). The results shown in figure 2 confirm this expectation and provide some additional useful evidence (these results are also used as an example in appendix B). As shown in the left panel, the most frequent outcome is not the most fit; the top two most frequent outcomes fall in the middle of the fitness distribution. Meanwhile, there is a strong and roughly proportional effect of mutation rate, as shown in the centre panel. The right panel confirms that this effect of mutation rate is not owing to confounding with selection coefficients, which are uncorrelated with the mutation rates.

In a well-known study of recurrent evolution, Rokyta *et al.* [59] carried out one-step adaptation 20 times in replicate populations of bacteriophage *ϕX174*, under conditions of adaptation from new mutations. They found that the most frequent change, repeated six times, was not the most fit, but rather the fourth most fit. These results were not in agreement with the model of Orr [79], which assumes uniform mutation, prompting the authors to seek a mutational explanation. They found that an origin-fixation model incorporating (i) measured selection coefficients, and (ii) a model of nucleotide mutation rates (from comparative data) performed better in predicting outcomes than Orr’s [79] model, which assumes homogeneity in mutation rates. Thus knowledge of mutation rates improved the predictability of adaptive outcomes.

**Figure 3.**
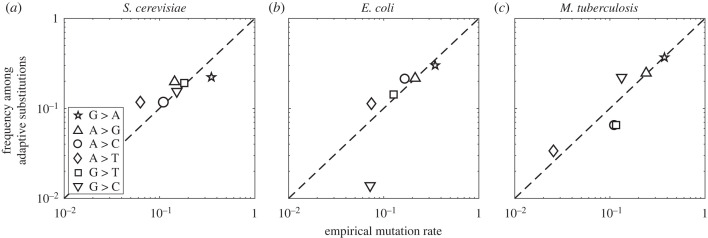
The frequency of nucleotide changes among adaptive substitutions as a function of the empirical mutation rate for (*a*) *S. cerevisiae*, (*b*) *E. coli* and (*c*) *M. tuberculosis*. The symbols correspond to the six different types of point mutations (inset in *a*). The dashed line shows *y* = *x*. Data from Cano *et al.* [56].

As explained in §2, Cano *et al.* [56] developed a method to capture the influence of the mutation spectrum with a single coefficient of mutational influence *β* that ranges from 0 (no influence) to 1 (proportional influence). They also applied this method to three datasets of adaptive amino acid substitutions, including substitutions implicated in natural adaptation of *Mycobacterium tuberculosis* to antibiotics, as well as laboratory adaptation of *E. coli* and *Saccharomyces cerevisiae* to environmental stress, using independently curated species-specific mutation spectra that describe the relative rates of the six possible nucleotide changes within double-stranded DNA. For each species, they found that *β* is close to 1 and significantly different from 0, indicating a proportional influence of the mutation spectrum. Moreover, they showed this was not just an effect of transition bias, but rather of the entire distribution of rates across the six types of single-nucleotide changes. Indeed, the frequencies of the six types of nucleotide changes among adaptive substitutions are strongly correlated with the independently curated species-specific mutation spectra (figure 3). The authors note that the three species differ in important population genetic conditions, such as mutation supply. Whereas *M. tuberculosis* has one of the lowest mutation rates of all bacteria [80] and is therefore likely to experience only limited clonal interference during adaptation to a new human host [81,82], *E. coli* and *S. cerevisiae* have relatively higher mutation rates [83,84] and often experience clonal interference in laboratory evolution experiments [85,86]. The results of Cano *et al.* [56] therefore provide empirical support for the theoretical result that mutation bias can influence adaptation across a broad range of population genetic conditions [29,57].

### (c) Scope of applicability

Now, having established causal agency and the potential for large effect-sizes, let us consider the scope and generality of this kind of cause-effect relationship. How widely can we expect it to apply? An ideal way to address this question would be to carry out a meta-analysis of published studies of adaptation. We would want to include in this analysis all of the relevant work, dividing it into experimental and natural adaptation, and perhaps considering other factors such as taxonomy and population size. At present, such an analysis would be quite difficult and would cover only a very minor fraction of the literature. The difficulties may be summarized as follows. In over a century of experimental studies of adaptation, the vast majority do not include a genetic analysis. Those with a genetic analysis typically implicate loci or alleles (e.g. involved in the adaptation of quantitative traits) without identifying specific mutations. The adaptation studies that implicate specific mutations (a tiny fraction of all adaptation studies) typically do not have sufficient replicates to support powerful tests, e.g. sometimes they are a one-off case [87]. In addition, most reports implicating adaptive mutations do not follow a rigorous standard for making this determination, so that mis-attributions are common [88], a serious problem given the prior expectation that non-adaptive changes will show effects of mutation biases. Furthermore, even in cases where adaptation can be traced confidently to specific mutations, we rarely have the kind of information on mutation biases and selection coefficients that would be needed to reach the conclusion that mutational effects are consequential once selection is taken into account.

The meta-analysis strategy of Stoltzfus & McCandlish [89], focused on transition-transversion bias among amino acid changes, was designed to maximize the use of available data given these difficulties. Briefly, it takes advantage of the following: (i) many contemporary studies of adaptation implicate specific amino acid changes, typically caused by single-nucleotide substitutions, doing so in a rigorous way based on verifying effects with genetic comparisons or engineering, (ii) for a broad range of taxa, nucleotide mutations show a bias towards transitions, typically two- to fourfold above null expectations [30,90], and (iii) experimental studies of mutational effects do not reveal any substantial tendency for transitions to be more benign than transversions [90,91], so that a reasonable null expectation for beneficial (or neutral) changes in the absence of mutation bias is a simple 1 : 2 ratio, given that there are twice as many possible transversions, as argued by Stoltzfus & McCandlish [89]. A substantial excess of transitions, e.g. a 1 : 1 or 2 : 1 ratio, would indicate an effect of mutation bias. Note that, in the literature of molecular evolution, it was long supposed that transitions are more conservative in their effects on proteins, as discussed by Stoltzfus & Norris [90]. However, this idea is not supported by systematic fitness measurements for amino acid-changing mutations, which show that transitions and transversions hardly differ at the upper end of the fitness distribution [90,91], though there may be some differences at the lower end, as argued by Lyons & Lauring [91].

On this basis, one may gather qualifying results and combine them, applying statistical tests for an excess of transitions relative to the 1 : 2 expectation. For instance, Meyer *et al.* [92] carried out replicate laboratory evolution experiments with bacteriophage *λ* under conditions that favour changes in the *J* gene, the product of which helps the virus target its bacterial host. Among 241 putatively adaptive changes, the ratio of transitions to transversions was 192 : 49, roughly eightfold higher than the 1 : 2 null expectation. The meta-analysis by Stoltzfus & McCandlish [89] covers experimental and natural adaptation using this approach, with the added safeguard that results are restricted to recurrent amino acid changes, i.e. their dataset is conditioned on parallel evolution. The experimental dataset covers five different experimental systems, the largest of which are the study of Meyer *et al.* [92] and the studies of Crill *et al.* [93] and Bull *et al.* [94] that uncovered numerous reversals and parallels in lines of *ϕX174* propagated through successive host reversals (between *E. coli* and *Salmonella typhimurium*). Combining the data from all five studies, Stoltzfus & McCandlish [89] find a highly significant 304 : 83 ratio of transitions to transversions among events of parallel adaptive amino acid changes.

Several subsequent studies have shown effects of transition-transversion bias. Sackman *et al.* [95] extended the earlier study of Rokyta *et al.* [59] by applying the same 20-replicate protocol to three additional types of phages, for a total of 80 adaptive changes. For each of the four phages, the most common variant to evolve was not the one with the largest fitness benefit. Out of 20 × 4 = 80 changes, the transition-transversion ratio was 74 : 6, a striking result. Likewise, Bertels *et al.* [96] observed a strong enrichment of transitions among adaptive mutations in propagation of HIV-1 in human T-cell lines, and Katz *et al.* [97] observed a bias towards transitions during adaptation of *E. coli* to long-term stationary phase.

What about adaptation in nature? The meta-analysis of Stoltzfus & McCandlish [89] includes data from 10 cases of natural adaptation traced to specific mutations, with results shown in table 1. For example, species such as monarch butterflies (*Danaus plexippus*) evolve resistance to cardiac glycosides by changes in the sodium pump ATP*α*1 [98–100], which not only allows them to eat *Apocynaceae*, but also to sequester the toxin in their tissues, making them noxious to predators. Other cases involved adaptation to natural or anthropogenic toxins (tetrodotoxin, insecticides, benzimidazole and the antiviral agent Ritonavir), altitude adaptation via haemoglobin changes, convergent foregut fermentation, trichromatic vision and echolocation. Combining the data from these cases of natural adaptation, Stoltzfus & McCandlish [89] uncovered a ratio of 132 transitions to 99 transversions (table 1)—a 2.7-fold enrichment over the null.

**Table 1.** Transition bias among natural parallelisms [89]. (For 10 different study systems implicating diverse taxa, the counts of parallel events are given for transitions (Ti) and transversions (Tv) (because this study is conditioned on parallelism, each type of change has at least two events). The results show a strong bias towards transitions. Note that some cases (marked by *) represent recent local adaptation of sub-populations to anthropogenic substances, while the rest refer to episodes of adaptation from the distant past.)

phenotype	taxon	target	Ti events		Tv events	
			counts	sum	counts	sum
insecticide resistance*	insecta	Rdl, Kdr, Ace	2, 2, 5, 2, 3	14	9, 2, 4	15
tetrodotoxin resistance	vertebrata	Na channels	2, 6, 3	11	2, 2, 2, 3, 3	12
dlycoside resistance	metazoa	Na^+^/K^+^-ATPase	4, 4, 2, 2	12	7, 2, 2, 4	15
herbicide resistance*	Poaceae	ACCase	5, 2	7	7, 2, 4, 5	18
altitude adaptation	Aves	*β*-haemoglobin	4, 13	17	2, 3, 2	7
trichromatic vision	vertebrata	opsins	2, 5	7	6, 4, 2	12
echolocation	mammalia	prestin	2, 2, 2	6	3, 2	5
growth in Ritonavir*	HIV1	protease	25, 7, 9	41	4	4
foregut fermentation	vertebrata	ribonucleases	2, 4, 4	10		0
benzimidazole resistance*	ascomycota	*β*-tubulin	7	7	5, 6	11
totals				132		99

Another example of transition-transversion bias in natural adaptation involves a very large set of resistance mutations identified clinically in the global human pathogen *M. tuberculosis*, which exhibits a strong mutation bias towards transitions [101] and evolves resistance to antibiotics exclusively through chromosomal mutations [102]. Examining two independently curated datasets, Payne *et al.* [103] uncovered transition-transversion ratios of 1755 : 1020 and 1771 : 900, a 3.4-fold and 3.9-fold enrichment over the null, respectively. They also took advantage of the special case of Met-to-Ile replacements, which can occur via one transition (ATG → ATA) and two transversions (ATG → ATT and ATG → ATC). Thus a 1 : 2 ratio is expected under the null hypothesis in which mutation bias has no effect. Instead, they observed ratios of 88 : 49 and 96 : 39 in the two datasets, roughly in fourfold excess of the null expectation.

What about other forms of mutation bias? In mammals and birds, mutation rates are elevated at cytosine-guanine dinucleotides (CpG) relative to other sequence contexts, owing to the effects of cytosine methylation on DNA damage and repair [104–106]. Genetic studies of high-altitude birds provided the first hints that this form of mutation bias may influence adaptation in nature, specifically the evolution of increased affinity of haemoglobin for oxygen, which is probably adaptive in hypoxic conditions and preferentially occurs via missense mutations at CpG dinucleotides [107,108]. Building off these observations, Storz *et al.* [109] systematically analysed the genetic sequences of haemoglobins in 35 matched, phylogenetically independent pairs of high- and low-altitude bird populations. Among the 35 pairs, they found 22 changes in oxygen affinity plausibly linked to altitude adaptation, implicating 10 different amino acid changes in haemoglobins. Of these 10 amino acid changes, six involved CpG mutations, whereas only one CpG mutation would be expected by chance, a significant excess. Thus, altitude adaptation in natural bird populations shows a significant enrichment of mutationally likely genetic changes, specifically mutations at CpG dinucleotides.

Taken together, the evidence summarized in this section provides robust support for a large and predictable influence of mutation biases on the changes involved in adaptation. The most common adaptive variants are often not the most fit, but the ones with the highest mutation rates. Quantitative biases in nucleotide mutation rates can have proportional effects, leading to a detailed match between the mutation spectrum and the spectrum of adaptive changes, and results from episodes of natural adaptation traced to the molecular level suggest a broad taxonomic scope.

## 4. Applications

Addressing ecological, agricultural and biomedical challenges often involves seeking to limit the reproduction of threatening biological agents such as microbial pathogens and parasites. Accordingly, understanding the evolutionary processes that give rise to problems of drug and pesticide resistance can lead to marked advancements in the agricultural and biomedical sciences. Extrapolating from its general use in evolutionary modelling, here we discuss how considerations of mutation-biased evolution shows tremendous potential in addressing challenges of widespread human concern, with a particular focus on evolutionary dynamics in somatic contexts such as cancer, drug and pesticide resistance and infectious disease.

### (a) Somatic evolution

Human somatic DNA mutates throughout adulthood in a manner that can cause disease, particularly as repeated rounds of genome replication in mitotically active cells provide opportunities for the emergence of mutant cells that have a replicative advantage, often to the detriment of the organism. Accounting for biases in mutation rates can provide improved insight into the evolutionary dynamics that occur among somatic cells [110]. In recent years, several studies exploring the evolutionary conversion of healthy somatic cells to cancer cells have shed valuable insights on the roles played by mutation biases [110,111]. These are typically characterized as so-called mutation signatures [112,113], which describe nucleotide mutation rates within a triplet context. When such signatures are constructed using DNA sequencing data from cancer cells, they simultaneously reflect mutation biases from endogenous sources such as DNA repair processes, as well as exogenous sources such as tobacco smoke [114]. Bioinformatic techniques can then be used to decompose this global mutation signature into underlying mutation signatures that can be attributed to these endogenous and exogenous sources [112]. For example, the mutational signature associated with APOBEC enzymes, which catalyse the deamination of cytosine bases, is a major contributor to mutational burden in head and neck squamous cell carcinomas [112]. A recent analysis of such mutations found that the relative importance of mutations for the cancer phenotype often differed from their prevalence, with some variants occurring infrequently despite being highly favoured by selection [115]. A more comprehensive analysis featuring 7815 cancer exomes identified dozens of highly statistically significant associations between cancer-driving mutations and specific mutational signatures such as those associated with environmental carcinogens and mutagenic enzyme activity [116]. The vast majority of these associations include deamination by APOBEC and deficiencies in proofreading and mismatch repair during replication. Intriguingly, this study also identified a negative association between tobacco smoke and the G12D substitution in KRAS; in other words, KRAS G12D is more common among the lung cancers of non-smokers [116]. Consistent with this finding, lung cancers harbouring the KRAS G12D substitution were recently associated with a lower tumour mutation burden [117,118], for which reason this mutation may serve as a negative biomarker for the success of immunotherapy. In addition to showing that the mutations most strongly favoured by selection are not necessarily the most prevalent among cancer patients, these findings suggest that mutational biases facilitate a link between the source of carcinogenesis and the predicted success of a given treatment.

What other factors may alter mutation rates in a manner that predictably influences the progression and treatability of cancer? Importantly, chemotherapy itself represents a source of mutagenesis, suggesting that attempts to treat cancer may inadvertently induce adaptive changes in the cancer that complicates further treatment options. For example, although mutations at residues 12 and 13 of the cell-signalling GTPase KRAS have a higher selective advantage, the Q61H mutation is common in colorectal cancers with resistance to the anti-EGFR antibody cetuximab [119], owing to a mutational signature associated with chemotherapy that elevates T > G transversions. Importantly, this work suggests that mutation signatures can serve as a basis for predicting the evolution of drug resistance in cancer patients. More recent investigation has also found that depending on the cancer type, the predominant driver mutations can arise from ‘actionable’ mutation signatures. In addition to tobacco, these drivers include mutations associated with exposure to ultraviolet light and endogenous processes associated with ageing [120]. By identifying specific causal factors underlying the likelihood of driver and drug-resistance mutations across different types of cancers, these findings provide a basis for predicting the efficacy of preventative and therapeutic strategies.

Finally, in tissues such as skin and blood, the relative over-proliferation of cell lineages with mutations conferring growth advantages is another medically important evolutionary process, and a target for prediction that may be informed by mutation rates. For instance, in the case of clonal haematopoesis, context-dependent nucleotide mutation rates play an important role in determining the prevalance of different variants [121]. The most frequent variant in the most frequently implicated gene, DNMT3a, is a CpG hotspot mutation changing Arg882 to histidine; but a change from Arg882 to cysteine, which occurs with a lower mutation rate, confers a larger growth advantage [121]. Similarly, a recent study of chronic myelogenous leukaemia found that for the tyrosine kinase inhibitor imatinib, epidemiological incidences of mutations conferring drug resistance are best predicted by the likelihood of the mutations rather than by their fitness effects [122]. Together, these results highlight mutation bias as an important predictor of somatic disease risk as well as drug resistance.

### (b) Resistance to biochemical agents

The evolution of resistance to drugs and host immunity represent substantial obstacles in the fight against disease. Accordingly, by providing insights on the processes underlying adaptive evolution, accounting for the combined roles of mutation and selection can improve our ability to understand and thus predict how resistance evolves among microbial pathogens. Specifically, are some mutational trajectories towards drug resistance enriched for higher-probability mutations than others, and can this information be used to fight infectious disease, in particular by tailoring treatment approaches that minimize the predicted likelihood of drug resistance? Recent work exploring large datasets of drug-resistance mutations has begun to shed light on these questions. For example, the study by Payne *et al.* [103] discussed in the previous section suggests the evolution of antibiotic resistance in *M. tuberculosis* is at least partially predictable, with some mutational paths towards resistance occurring more frequently than others depending on the relative abundance of transition mutations. Moving forward, it will be greatly informative to comprehensively characterize mutational paths towards resistance in a greater diversity of infectious pathogens and across a wide panel of antibiotics. By identifying drugs or drug cocktails for which mutational paths towards resistance tend to be relatively depleted of high-probability mutations, it may be possible to employ treatment regimens that minimize the predicted likelihood of evolved resistance, enabling treatments with longer-lasting effectiveness.

Besides transition-transversion bias, how else might biases in mutation rate guide the evolution of drug resistance? The idea that the most mutationally probable changes are not necessarily the most strongly favoured by selection implies the existence of potential mutations that would be highly adaptive but whose rates of occurrence are negligible. Accordingly, by altering the relative rates of mutation types, changes in the sources of mutation may promote adaptation by enhancing access to otherwise-rare beneficial mutations [50,53]. In one recent example, point mutations in a DNA topoisomerase gene, which is important for relieving topological stress in DNA strands, were reported to introduce mutation hotspots that result in new adaptive paths towards antibiotic resistance in *E. coli* [123]. Although the relevance to infectious pathogens remains unclear, these findings highlight a promising approach towards identifying new potential mutational paths to the evolution of antibiotic resistance. In particular, future work may be able to determine whether mutations in DNA maintenance or repair genes shift the mutation spectrum in a manner that promotes drug resistance evolution in pathogenic bacteria. Granting such insights, we anticipate the potential for bacterial genotyping as a predictor for the likelihood of evolved resistance to specific classes of antibiotic.

As with antibiotics, the widespread use of pesticides and fungicides in agriculture can select for the evolution of resistance, which has been reported in hundreds of species [124,125]. Similar to many examples discussed in the previous sections, mutation biases have been implicated in instances of insecticide, fungicide and herbicide resistance (table 1) [89], suggesting a broad range of potential agricultural applications for incorporating mutation bias into evolutionary forecasting. In addition to transition-transversion biases, how else might mutation biases improve the predictability of resistance evolution? To address this question, we consider examples of natural mutators. Powdery mildews are fungal plant pathogens that belong to the genera *Erysiphe* and *Blumeria* and represent a major agricultural threat [126]. These taxa have undergone extensive loss of DNA mismatch repair genes throughout their evolutionary history, leading to rapid, mutation-biased genome evolution [73]. Importantly, heavy use of fungicide has been reported to accelerate the evolution of resistance in these taxa [126]. Whether variation in the mismatch repair pathway predictably alters the likelihood of resistance evolution remains unclear. However, the number of mismatch repair genes lost during evolution varies greatly across taxa and correlates with nucleotide substitution and composition biases [73], which raises the interesting possibility that the tendency for the genomic changes that facilitate fungicide resistance might also correlate with the loss of these genes. It would be fascinating to address this possibility in future work, in particular by interrogating the mutation spectra produced from targeted disruption of mismatch repair for their tendencies towards fungicide resistance.

### (c) Infectious disease

The COVID-19 pandemic represents an exceptional case study in the importance of forecasting evolutionary trajectories among both real and potential pathogens. Since the start of the pandemic, scientists and medical professionals have sought to understand the mechanisms underlying both disease severity and viral evolution, with the goal of maximizing mitigation efforts and vaccine effectiveness. Toward this end, numerous investigations of SARS-CoV-2 genomes have identified mutation biases strongly favouring uracil content, with potential implications ranging from vaccine design to personalized therapies and the emergence of new viral variants. For example, Rice *et al.* [127] recently reported that although mutation bias strongly favours U content, selection largely occurs against U content, which raises the question about how informative this mutation bias may be towards predicting adaptive changes. On the other hand, a strong C-to-U mutation bias was more recently reported to drive the diversification of CD8+ T-cell epitopes and the depletion of proline residues, which has been suggested to compromise T-cell immunity among individuals carrying the human leucocyte antigen B7 serotype [128]. Because host immunity represents a strong source of selection pressure on viral replication, the C-to-U bias may be helping to sustain high COVID-19 infection rates by facilitating immunity evasion within at least a subset of the population. Thus, in addition to the evolution of drug resistance as described above, mutation bias may shape the evolution of viral pathogens in a manner that predictably disrupts host immunity.

Given the abundance of recent changes in the spike protein [129], it may be possible to draw statistical inferences about whether, and to what extent, recent adaptations in SARS-CoV-2 are mutation-biased. This will greatly inform our ability to develop and implement accurate pandemic forecasting. In particular, the most commonly observed adaptive mutations are not necessarily the most fit. Accordingly, if the recent evolution of SARS-CoV-2 has been largely determined by amino acid changes that are mutationally favoured but selectively suboptimal, then there may exist adaptive ‘jackpot’ mutations that have yet to be sampled. In this case, a prolonged high rate of infections could be expected to enable the eventual occurrence of low-probability mutations that substantially enhance viral transmission. This scenario seems consistent with the recurrent emergence of increasingly transmissible variants. On the other hand, high COVID-19 infection rates raise the question of whether the evolution of SARS-CoV-2 is mutation-limited, especially given the ability of new variants to spread between geographical regions and populations. In either case, the rapid accumulation of amino acid replacements provides a considerable sample of empirical data. These data could be combined with estimates of mutation rates in order to determine whether recent or future emergence of increasingly transmissible variants are driven by systematic relationships between mutation rates and fitness effects.

In addition to SARS-CoV-2, the rapid pace of adaptive evolution has made some pathogens such as HIV notorious for their ability to evade our efforts to employ treatments and vaccinations with long-term efficacy. As a retrovirus, HIV requires reverse transcriptase to infect hosts, and numerous reverse transcriptase mutations can confer resistance to reverse transcriptase inhibitors that are used to treat HIV infection. Importantly, owing to a bias favouring the G-to-A transition mutation, the resistance-conferring M184I replacement in reverse transcriptase was found to occur more readily than M184V, despite the latter conferring greater replicative fitness [130]. Consistent with this finding, theoretical modelling has implicated G-to-A mutations, mediated by the APOBEC family of host deaminases, in the evolution of drug resistance in HIV [131].

How might such biases aid in the predictability of HIV evolution? Recent work suggests that instances of parallel evolution serve as a promising source of insight on this question. In particular, the relative number of independent occurrences of a given type of evolutionary change reflects its underlying probability: if one of two types of evolutionary change has a twofold greater probability of occurring, it can be expected to occur twice as often in independent lineages. Since the chance of parallel or repeated evolution increases with greater variance in mutation rates (see appendix B), mutation biases raise the probability of particular types of evolutionary change. Consistent with these theoretical expectations, a long-term evolution experiment involving the continued passaging of HIV in human T-cells revealed numerous instances of parallel changes, characterized by a strong bias for G-to-A transitions [96]. Unfortunately, because long-term evolution is bound to involve the accumulation of both adaptive and neutral changes, such experiments pose the challenge of disentangling the roles of mutation and selection.

To overcome this difficulty, deep mutational scanning can be used to isolate the functional effects of massive numbers of individual mutations. For example, Haddox *et al.* [132] used deep mutational scanning to characterize the amino acid preferences at every site in the envelope proteins from two HIV lineages. Results from such experiments can be combined with measures of mutation rates to generate pairwise estimates of rate and fitness effect for large numbers of potential mutations. Such pairwise estimates enable the prediction of likely sequence changes during evolution, since the rate of such changes are jointly proportional to both mutation rate and selection coefficient (equation (2.1)). Finally, given its rapid rate of evolution, long-term evolution experiments with HIV such as the one performed by Bertels *et al.* [96] provide a wealth of empirical sequence changes for testing and refining evolutionary predictions. By identifying adaptive paths involving low-probability mutations, such an approach could potentially uncover new drug and vaccine targets that minimize the likelihood of evolved resistance, leading to treatment regimens with longer-term effectiveness.

## 5. Challenges

Theory and empirical evidence indicate that mutation biases can have predictable effects on the genetic changes fixed in adaptation under a broad range of population-genetic conditions. In the context of research on the predictability of evolution, the obvious application of these results is simply to absorb the science that is already well established—nucleotide substitution biases shape short-term adaptation—and apply that by using available information on the mutation spectrum.

Beyond these obvious applications, what further gains would be possible with new technology or a shift in resources and attention? In this section, we suggest specific areas in which a stronger focus on effects of biases in variation might yield substantial gains, including (i) improving measurements of basic quantities, (ii) expanding our attention beyond nucleotide substitution biases, and (iii) assessing the predictability of mutational effects across diverse conditions and timescales.

### (a) Expanding coverage of basic measurements

The results reported above show the value of obtaining fundamental measurements of the following three quantities, for each possible outcome: selection coefficient (*s*), mutation rate (*μ*) and frequency of evolution (*f*). In particular, the example of Maclean *et al.* [62], as employed in figure 2 and appendix B, shows that such data are extraordinarily valuable, yet this case is small—just 11 variants—and we know of no other comparable dataset.

More commonly, we have access to individual measures of functional effects via deep mutational scanning, but no individual mutation rates, which are instead represented by a model of average rates for classes, e.g. a model of two rates for transitions and transversions, or a model of six types of nucleotide substitutions. Yet, as explained in appendix B, prediction will always suffer when models of average rates are used. Direct and indirect evidence indicates individual rates have a large amount of variance (i.e. useful information) that simplified models of mutation rates simply do not capture, e.g. Maclean *et al.* [62] find a 30-fold range in mutation rates for just 11 nucleotide substitution mutations in the same gene; Hodgkinson & Eyre-Walker [67] use a comparative method to estimate that a triplet context model captures only about one-third of the actual variance in mutation rates.

The technical barriers to addressing this rather stunning deficit are rapidly disappearing. Until recently, methods for measuring mutation rates dated from the 1940s and were used infrequently [133]. However, new methods for identifying and tracking mutations are now appearing rapidly, including methods based on real-time visualization [134], and methods specifically designed to measure mutation rates accurately in deep sequencing experiments [135,136]. We note that, if estimates of *μ*, *s* and *f* are used to interrogate their relationships, it is imperative to ensure that the estimates are unbiased with respect to these relationships. For instance, some methods used to study somatic evolution, e.g. clonal haematopoesis in Watson *et al.* [121], infer both *μ* and *s* from a joint distribution of population frequency and somatic prevalence (measures obviously related to *f*), and this raises the question of whether they are subject to correlated errors, e.g. if under-estimation of *s* induces over-estimation of *μ*.

We look forward to a future in which quantitative scientists have access to well defined sets of fundamental measurements for diverse model systems in somatic evolution, the emergence of resistance to toxins, and the adaptive evolution of infectious agents exploiting host resources.

### (b) Exploring diverse sources of variational bias

Most approaches to analysis and modelling that incorporate rates for nucleotide substitution mutations use a simplified model, e.g. a two-parameter model (i.e. transitions and transversions) or a six-parameter model. However, as noted above, such models capture only a minority of the variance in individual rates [67]. This is particularly important given the common observation that adaptive outcomes are often highly enriched for a few high-rate mutations that happen to be favourable. This suggests an importance for improving models for mutation hotspots, a topic that is rarely treated (e.g. [137]). In addition, one must not overlook the possibility of highly consequential correlations between mutational and functional features of genomes, e.g. as in Monroe *et al.* [76]. Such correlations are important to consider whether one thinks of them as evolved features (as argued by [76]) or coincidences, e.g. Monroe *et al.* [138] find that, in prokaryotic genomes, transcription-replication collisions result in some very specific and large effects, including an orientation-dependent fourfold increase in point mutations affecting promoters, mostly due to *T* → *C* mutations at position-7 relative to the start of transcription.

Although much work remains to be done in terms of basic measurements and models regarding single-nucleotide mutations, there is a far larger universe of possible mutations to explore, including multi-nucleotide mutations, compound changes affecting dispersed sites, microindels (very small insertions and deletions), the expansions and contractions of highly variable repeat loci, segmental duplications, transposable element insertions, inversions, and chromosome fission and fusion. A quantitative overview of this universe of mutations is given in (Stoltzfus [33], app. B). Within each of these categories, the distribution of individual mutation rates will reflect (i) the immediate sequence context [112,139], (ii) the regional chromosomal environment including local states of expression and chromatinization [32,140], (iii) aspects of the state of the cell reflecting age, DNA repair activities and cell-cycle state (e.g. differences in nucleotide precursors, repair enzymes or reactive oxygen species) [141–143], and (iv) broad environmental factors such as ambient radiation (e.g. exposure to ultraviolet light) and temperature [68,69].

Opportunities to improve prediction in this regard arise most obviously when, for some specific prediction problem, evolution commonly involves mutations other than nucleotide substitutions (and they also arise, less obviously, when such mutations are probable under some conditions but are not observed). For instance, segmental duplications occur commonly in experimental yeast adaptation (e.g. [144]), transposable element insertions are commonly implicated in local adaptation of bacteria in nature (e.g. [145]), and highly variable short-tandem-repeat loci have been implicated in cases of short-term adaptation such as the domestication of dogs [146]. A case of particular interest are multi-nucleotide changes to codons [147], which have been observed in studies of cancer [148], developmental disorders [149], SARS-CoV2 [150,151], and resistance to antimicrobials, e.g. in *M. tuberculosis* [152]. Such mutations are a target of opportunity given the kind of information already available, namely: (i) deep mutational scanning studies, which cover the amino acid changes that occur by double- and triple-nucleotide changes to codons, and (ii) prior information on the underlying rate for tandem double or triple mutations in eukaryotes, which appears to be (in total) about two or three orders of magnitude less than the total rate of single-nucleotide changes [147].

Finally, we stress that the literature on natural and experimental evolution covers a variety of phenomena—under the headings of predictability, contingency (repeatability), constraints, genotype–phenotype maps, and so on—that are not usually associated with the concept of mutation bias but which are subject to the same rules of population genetics as mutational effects under a scheme of aggregation (appendix B). For instance, the genetic code is a genotype–phenotype map dictating that there is one single-nucleotide mutational path from Met (ATG) to Val (GTG), two paths from Met to Leu (TTG, CTG) and three paths from Met to Ile (ATT, ATC and ATA). The biases induced by this mapping are not the same thing as mutation biases in the narrow sense of biases imposed by the mechanism of mutagenesis: instead, they are induced by an asymmetric mapping of genetic changes into a phenotype space. Nevertheless, from the perspective of understanding effects of biases in the introduction of variation, a scheme of aggregation that imposes twofold or threefold biases has the same impact as a twofold or threefold mutation bias. Likewise, when the mutational target size of a trait or the mutational accessibility of an alternative phenotype is identified [48,63,153–155], this corresponds to a scheme of aggregation over elementary mutational events. For instance, the series of studies from [155,156] dissecting the emergence of the wrinkly spreader phenotype in *P. fluorescens* provides a detailed look at asymmetries in the mutational accessibility of an alternative phenotype. Analyses of genotype–phenotype maps in a wide diversity of biological systems reveal that such asymmetries are common [157]. In long-term evolution, the phenotypes that have more genotypes are on the whole more ‘findable’ [51,158,159]. Understanding the extent to which these biases have predictable effects on the genetic changes fixed in adaptation is an important direction for future research, i.e. the challenge is to measure the predictive accuracy of different kinds of aggregation (and some guidance for doing so is provided in appendix B).

### (c) Considering diverse conditions and timescales

The most robust empirical and theoretical results available today focus on short-term or one-step adaptation, and the effects are best understood for the case of mutation-limited conditions, although we are beginning to get a clear sense of what happens as mutation supply increases and clonal interference becomes common in finite spaces [29,56,58] or infinite ones (appendix B, [57]). These results are relevant to many challenges in prediction, as we have argued above, e.g. antibiotic resistance. However, a challenge for future research is to expand the consideration of mutational effects to cover longer timescales and a greater diversity of contexts, including evolution from standing variation and synthetic evolution. Attempts to predict long-term effects of mutation bias, for instance, can take advantage of limited theory currently available on how mutation bias influences multi-step trajectories to adaptation [49–53].

Regarding evolution from standing genetic variation, when multiple beneficial alleles are present in numbers high enough to escape random loss, the most fit allele typically wins [63]. This appears to leave no room for effects of mutation bias, but actually it just pushes the question of origination biases into a different realm, where the primary question concerns how the distribution of standing variation is shaped by tendencies of mutation. For instance, the rate of length changes in short tandem repeat loci is so high that the vast majority of such loci will exhibit standing variation for length in a moderately sized population, and this is relevant for cases such as short-term evolution of gene expression [160].

Finally, mutation bias may improve evolutionary forecasting in synthetic evolution [161], such as in laboratory evolution experiments with genomically recoded organisms [162–165] or engineered mutagenesis mechanisms [166]. For instance, a directed evolution technique called Orthorep uses an orthogonal DNA polymerase to introduce mutations to plasmid-borne target genes [166]. The polymerase’s mutation spectrum is heavily biased towards G : C → A : T transversions [167], which may influence evolutionary outcomes, such as the primary and promiscuous functions of enzymes [168]. More broadly, synthetic evolution provides a useful testbed for mutation-biased adaptation theory, as the mutation spectrum can be manipulated under controlled laboratory conditions, and evolutionary outcomes can be quantified with DNA sequencing and phenotypic assays.

## 6. Conclusion

We have presented theoretical arguments and empirical evidence that mutation bias can have predictable effects on the genetic changes fixed in adaptation. In studies of adaptation in diverse contexts, where fitness effects have been measured, it is regularly observed that the most common outcome is not the most fit: instead, it is often a beneficial variant with an extreme rate of mutational origin. More generally, the spectrum of changes observed in adaptation reflects the mutation spectrum. Sometimes this effect can be quite strong, even proportional. The study of mutation-biased adaptation has achieved some degree of quantitative and theoretical sophistication, although much remains to be determined about factors such as the influence of population-genetic conditions, and the scope of applicability in natural adaptation.

On this basis, we can make a strong argument that knowledge of mutation bias can be used to improve evolutionary forecasting. We have highlighted applications where we think this approach may prove particularly valuable, in relation to somatic evolution, resistance to toxins and the adaptation of infectious agents to make use of host resources. Our hope is that this review will serve as a useful source of guidance for those implementing approaches to prediction, and that the information contained in it will be quickly eclipsed by more diverse, general and precise results.

## Figures and Tables

**Figure 4.**
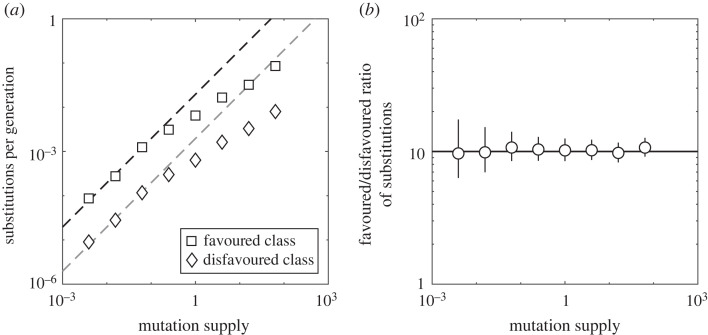
Simulation of the infinite sites model. Two classes of mutations were simulated, one occurring with 10 times the frequency of the other. The fitness effects of the two classes of mutations have the same exponential distribution and they combine additively. (*a*) The number of fixed mutations per generation. The dashed lines show the expectation under the origin-fixation regime (i.e. no clonal interference) for the favoured class and the disfavoured class in black and grey, respectively. Note the departure of the observed number of fixed mutations from the origin-fixation expectation as 2*Nμ* approaches 1, as the fixation of new mutations is slowed down by clonal interference. (*b*) The ratio of the favoured and disfavoured class among the fixed mutations. Vertical lines represent 95% confidence intervals estimated using the Poisson distribution. Analogous results for one-step adaptation are shown in the electronic supplementary material, figure S1.

## Data Availability

Data for figure 1 are provided as the electronic supplementary material [169]. Data for figure 2 are provided in Maclean *et al.* [62]; data for figure 3 are provided in Cano *et al.* [49]; Data for table 1 are provided in Stoltzfus & McCandlish [89]. Data and code for figure 4 are provided in the Github repository indicated in the figure caption.

## Appendix A. Mutational biases in models with finite and infinite sites

Consider the case where there are *m* possible beneficial mutations, and the *i*th beneficial mutation has selection coefficient *s*_*i*_ and mutation rate *μ*_*i*_. We assume that the *s*_*i*_’s are drawn independently from the same distribution of mutational effects on fitness. For a population of size *N*, under what circumstances do the *μ*_*i*_’s influence which of these *m* mutations will be the first to reach fixation?

To gain some intuition for this question it is helpful to consider two limiting situations:

(i) in the limit of very large mutation supply, in particular if *Nμ*_*i*_ ≫ 1 for all *μ*_*i*_, i=1,…,m, all possible beneficial mutations are introduced into the population in each generation. These mutations will all compete with each other and, assuming that mutation rates are small relative to selection coefficients, ultimately the fittest of the *m* mutations will go to fixation. To see the consequences of this result, consider single-nucleotide substitutions, which can be classified as either transitions or transversions. Because there are twice as many transversions than transitions, the fittest variant, that is the one that will eventually go to fixation, has two times the probability of being a transversion than being a transition. Thus, under these conditions, the expected transition : transversion ratio among adaptive substitutions would be 1 : 2, regardless of whether transitions arise at a higher rate; and
(ii) now let us consider the limit where all the *Nμ*_*i*_ are small (specifically, assume $\sum_{i}N\mu_{i}s_{i}\ll \min_{i}s_{i}/\log N$, so that the first beneficial mutation to become established in the population will typically have sufficient time to reach fixation before the next beneficial mutation is able to become established, [54]). In this setting, the probability that a mutation will be the first to go to fixation is proportional to both its mutation rate and its selection coefficient (equation (2.1)), so that all other things being equal, we expect that classes of mutation with high mutation rates, such as transitions, will be over-represented among fixed mutations. For instance, if the mutation rate for individual transitions is *κ* times the mutation rate for individual transversions (*μ*_*i*_/*μ*_*j*_ = *κ* if *i* is a transition and *j* is a transversion), then we expect a transition : transversion ratio of *κ* : 2 among fixed mutations.

Thus, broadly speaking, mutational biases will tend to have a stronger influence on molecular adaptation when the beneficial mutation supply is low, because in this regime the first beneficial mutation that becomes established in the population is likely to go to fixation rather than the fittest possible mutation, and the waiting time until a mutation becomes established is inversely proportional to its mutation rate.

Another common class of models are the infinite sites models, where we assume that each new mutation has a selection coefficient that is drawn independently from some distribution of fitness effects. In this class of models, if different types of mutations share the same distribution of fitness effects, then the relative proportions of different types of mutations among fixed mutations always varies in a manner directly proportional to the mutational bias. For example, if we consider transitions and transversions, for each selection coefficient *s*, the transitions : transversion ratio among mutations with that selection coefficient is *κ* : 2. Thus no matter how competition between co-segregating mutations alters the distribution of fixed selection coefficients relative to the overall distribution of fitness effects, the expected transition : transversion ratio among fixed mutations will always be *κ* : 2. The results of this intuition are shown graphically using evolutionary simulations in figure 4. Even though we see the effects of competition between multiple adaptive mutations as a departures from the origin-fixation expectation that sets in at a total mutation supply 2*Nμ* ≈ 1 (figure 4), the ratios of fixed mutations are proportional to the introduction rates regardless of the value of the mutation supply (figure 4). In the more general case of infinite sites models for mutational types that do not share the same distribution of fitness effects, the strict proportionality with mutation rates need not hold, however a similar intuition applies in that we can consider the relative proportion of each mutational type for each possible selection coefficient *s*, and then the relative frequencies of fixed mutations can be determined by averaging these proportions over the characteristic distribution of selection coefficients [55] fixed during adaptation.

The simulation code is available in a GitHub repository at https://github.com/alejvcano/infiniteSites.

## Appendix B. Quantifying contributions of mutation and selection to repeatability

How do mutation and selection contribute to the predictability of evolution? Can we partition their effects? One sense of predictability is repeatability, the chance that the outcome of evolution will match what we have seen before. Let us define repeatability as the chance of parallel evolution between a pair of trials. If we have *n* elementary outcomes, each happening with some probability *p*_*i*_, then repeatability *P*_para_ is the sum of squares of *p*_*i*_:

B1 $$P_{\mathrm{para}}=\sum_{i=1}^{n}p_{i}^{2}.$$

This is equivalent to the measure known as Simpson’s index *S*(***p***), where by ***p*** we denote the vector of *p*_*i*_’s (analogously, we use bold symbols to denote vectors of variables in the following). Simpson’s index has a simple relationship to the variance Var(p) or coefficient of variation *c*(***p***) of the distribution of elementary probabilities:

B2 $$P_{\mathrm{para}}=S(p)=n\text{Var}(p)+\frac{1}{n}=\frac{c{\left(p\right)}^{2}+1}{n}.$$

Under uniformity, Var(p)=c(p)=0, and *P*_para_ = 1/*n*. The greater the variance in probabilities, the greater the chance of parallelism. Any factor that increases variance, increases parallelism. Likewise, any approach to prediction that ignores variance, e.g. by aggregating outcomes into classes whose members are assigned the average behaviour of the class, will underestimate parallelism.

One of the ways to quantify the effect of heterogeneity is to compute an effective number of options with the same chance of recurrence under uniformity, equal to the inverse of the probability of parallelism, i.e. *n*_*e*_ = 1/*P*_para_, comparing that to *n*. If one state has *p* = 1 and the others have *p* = 0, then repeatability is 1, and *n*_*e*_ = 1. If all states are equally likely, then *p*_*i*_ = 1/*n* for all *i*, *n*_*e*_ = *n* and the repeatability is $\sum {\left(1/n\right)}^{2}=1/n$. From the 20 replicates of Rokyta *et al.* [59], the counts ranked by selection coefficient are ***k*** = [1, 5, 3, 6, 1, 1, 1, 1, 1], thus repeatability is $\sum_{i}{\left(k_{i}/20\right)}^{2}=0.19$, and *n*_*e*_ = 5.2, i.e. the effect of heterogeneity in *p*_*i*_’s is like reducing the choices from 9 to 5.2. Note this calculation ignores sampling error by treating the observed frequency *f*_*i*_ as the true probability *p*_*i*_.

How could we partition repeatability to effects of mutation and selection? This is possible for the special case of origin-fixation dynamics [47]. For event *i*, an origin-fixation process specifies a rate *Nμ*_*i*_*π*_*i*_, with *π*_*i*_ being the fixation probability of the event, so that its application in the current context means that *p*_*i*_ ∝ *μ*_*i*_*π*_*i*_. Then the chance of parallelism is given by

B3 $$P_{\mathrm{para}}=\frac{\left(c{\left(\mu \right)}^{2}+1)(c{\left(\pi \right)}^{2}+1\right)}{n},$$

if we can assume that covariance of ***μ*** and ***π***, as well as the covariance of the squares of *μ*_*i*_’s and squares of *π*_*i*_’s is 0. In general, the fixation probability *π*( · ) is a function of *s*, the selection coefficient, and *N*, population size [60]. However, under strong selection weak mutation conditions *π*(*s*, *N*) ≈ 2*s* [42], and this leads directly to the result of Chevin *et al.* [61]:

B4 $$P_{\mathrm{para}}\approx \frac{\left(c{\left(\mu \right)}^{2}+1)(c{\left(s\right)}^{2}+1\right)}{n}.$$

Consider some applications of equation (B 3). For the results of Rokyta *et al.* [59], *c*(***π***)^2^ + 1 = 1.086 for the probabilities of fixation computed from the reported selection coefficients, and *c*(***μ***)^2^ + 1 = 1.33 for mutation rates (given the model described by the authors), so mutational heterogeneity contributes slightly more. For the 11 variants from Maclean *et al.* [62] with known mutation rates, *c*(***μ***)^2^ + 1 = 7.93 and *c*(***π***)^2^ + 1 = 1.05, so mutational heterogeneity is contributing much more. This disparity reflects a 30-fold variability in mutation rates, but only about twofold range in probabilities of fixation, given that all the resistant variants have large selection coefficients (because *π*(*s*, *N*) ≈ 2*s* does not apply for large *s*, we must use equation (B 3) instead of (B 4)). Note that this framework does not fully partition the effects of mutation and selection, as these factors are conflated in determining *n*, which in practice reflects the number of mutations that are both sufficiently beneficial and sufficiently mutationally likely to have an appreciable chance of being detected.

Finally, it is of interest to consider repeatability when outcomes are aggregated. In the case of predicting phenotypes, a genotype–phenotype map is used to assign a phenotype to each of the *n* elementary outcomes. Alternatively, the categories could be defined by genes [63,64], pathways, or gene ontology (GO) categories, as per Tenaillon *et al.* [65]. Suppose that the *n* elementary outcomes are assigned fully to mutually exclusive groups of size *m*_1_, *m*_2_, …*m*_ℓ_ by a function *f* such that *f*(*i*) = *j* when outcome *i* is in group *j*, and suppose further that *f*( · ) assigns outcomes randomly. Then (as given in the mathematical appendix), the expected probability *P*_gpara_ that two outcomes of evolution are in the same group is:

B5 $$\begin{array}{rl} EP_{\mathrm{gpara}} & =\sum_{\;j=1}^{\ell }\left(\sum_{i|f\;(i)=j}p_{i}^{2}\right)=\frac{n}{n-1}\left(S(p)+S(g)-S(p)S(g)-\frac{1}{n}\right)\\ & \approx S(p)+S(g)-S(p)S(g), \end{array}$$

where *S*(***p***) is Simpson’s index over *n* elementary outcomes and $S(g)=\sum_{\;j=1}^{\ell }{\left(m_{j}/n\right)}^{2}$ is Simpson’s index of the partition into ℓ groups (and where the approximation is valid for large *n*). The effect of aggregation is always to increase parallelism. Note that equation (B 5) is symmetric in *S*(***p***) and *S*(***g***), so that the effect of the probability distribution for elementary events is the same as the effect of the probability distribution of categories. The use of this formula is that the prediction success of a concrete scheme of aggregation (e.g. GO categories) can be compared to the baseline expectation for a random aggregation with the same distribution of category sizes.
